# Understanding determinants of patients’ decisions to attend their family physician and to take antibiotics for upper respiratory tract infections: a qualitative descriptive study

**DOI:** 10.1186/s12875-020-01196-9

**Published:** 2020-06-24

**Authors:** Sameh Mortazhejri, Andrea M. Patey, Dawn Stacey, R. Sacha Bhatia, Alykhan Abdulla, Jeremy M. Grimshaw

**Affiliations:** 1 School of Epidemiology and Public Health, University of Ottawa, Ottawa, Canada; 2 Clinical Epidemiology Program, Ottawa Hospital Research Institute, Ottawa, Canada; 3 Faculty of Health Sciences, University of Ottawa, Ottawa, Canada; 4 Institute for Health System Solutions and Virtual Care, Women’s College Hospital, Toronto, Canada; 5 Institute of Health Policy, Management and Evaluation, University of Toronto, Toronto, Canada; 6 The Kingsway Health Centre, Manotick, Canada; 7 Faculty of Medicine, University of Ottawa, Ottawa, Canada

**Keywords:** Coping strategy, Illness representation, Self regulation model, Semi-structured interview, Upper respiratory tract infection

## Abstract

**Background:**

Although antibiotics have little or no benefit for most upper respiratory tract infections (URTIs), they continue to be prescribed frequently in primary care. Physicians perceive that patients’ expectations influence their antibiotic prescribing practice; however, not all patients seek antibiotic treatment despite having similar symptoms. In this study, we explored patients’ views about URTIs, and the ways patients manage them (including attendance in primary care and taking antibiotics).

**Methods:**

Using a qualitative descriptive design, adult English-speaking individuals at a Canadian health center were recruited through convenient sampling. The participants were interviewed using semi-structured interview guide based on the Common Sense-Self-Regulation Model (CS-SRM). The interviews were transcribed verbatim and coded according to CS-SRM dimensions (illness representations, coping strategies). Sampling continued until thematic saturation was achieved. Thematic analysis related to the dimensions of CS-SRM was applied.

**Results:**

Generally, participants had accurate perception about the symptoms of URTIs, as well as how to prevent and manage them. However, some participants revealed misconceptions about the causes of URTIs. Almost all participants mentioned that they only visited their doctor if their symptoms got progressively worse and they could no longer self-manage the symptoms. When visiting a doctor, most participants reported that they did not seek antibiotics. They expected to receive an examination and an explanation for their symptoms.

**Conclusion:**

Our participants reported good understanding regarding the likely lack of benefit from antibiotics for URTIs. Developing interventions that specifically help patients discuss their concerns with their physicians, instead of providing more education to public may help in reducing the use of unnecessary antibiotics.

## Background

Upper respiratory tract infections (URTIs) are one of the most common reasons to visit a doctor globally [1], but not all patients with similar symptoms see their doctor. Differences in ways people perceive their symptoms may account for some of this variation [2, 3]. Many primary care visits by patients with URTIs in Canada result in an antibiotic prescription [4]. Physicians are more likely to prescribe antibiotics if they believe that their patients expect them [5, 6]. Furthermore, studies report that physicians assume that prescribing antibiotics will lead to more satisfaction in patients [7–9]. However, physicians’ perceptions about their patients’ wishes do not always reflect patient expectations [5, 9–11]. Further, there is only limited evidence that some patients put pressure on their doctors to prescribe antibiotics directly by asking them or indirectly by the way they present their chief complaints [7, 12].

As patients are the end users of antibiotics, they play an important role in reducing over-prescription of antibiotics [8]. However, studies report that there are still misconceptions about the effectiveness of antibiotics for URTIs among patients [13, 14]. Social cognitive models can help us understand factors that may influence patients’ health-related behaviors [15]. They can help us to understand why some patients with URTIs visit their doctors to ask for antibiotics, while others manage it themselves. This study aimed to investigate patients’ expectations and beliefs about managing URTIs using the Common Sense-Self-Regulation Model (CS-SRM) [16].

## Common sense-self-regulation model

The CS-SRM is a model which attempts to explain how individuals’ perceptions and beliefs about an illness influence their behaviors toward that illness. This model can help us understand why some patients with URTIs go to doctors and ask for antibiotics, while the others prefer to manage it themselves [16].

According to CS-SRM, people develop beliefs and emotions about their illnesses (illness representations) relating to two major categories: *cognitive illness representations*, and *emotional illness representations*. Cognitive illness representations are further categorized into *identity, cause, timeline, consequences, curability/controllability* and *prevention* [16–19]. Individuals develop a set of coping strategies to cope with these illness representations [16, 20]. Coping strategies are often appraised and updated by the individuals based on success or failure in dealing with previous episodes of an illness. Each new experience is accompanied by additional information from other sources such as cultural knowledge of an illness (e.g. from media) or significant authorities like parents or doctors, resulting in the development of new illness representations. These new representations may consequently lead to new sets of coping strategies. The model is dynamic and is based on continuous feedback between different elements [16, 20].

While the CS-SRM model has mainly been applied to study chronic diseases (e.g. diabetes, Alzheimer’s Disease, epilepsy, asthma) [19, 21–23], it has been used to explore how general public in the UK managed their respiratory tract infections [24]. URTIs have an acute course and are typically resolved within a few days but people can experience them many times during their lifetime, which may be similar to symptom presentation of some chronic illnesses. Therefore, using CS-SRM to understand patients’ perceptions about URTIs and their coping strategies may prove insightful. The use of theory provides a framework within which the researchers can understand and analyse different aspects of data beyond their personal insights. It also increases the robustness and relevance of the findings and enhances the appropriate design of the interventions [25–27].

In this study, we aimed to explore how individuals perceived URTIs and how their perceptions influenced their management of URTIs (including self-management, primary care consultation and antibiotic use).

## Methods

We conducted a qualitative descriptive study [28] using semi-structured interviews based on CS-SRM. The study was approved by the Ottawa Health Science Network Research Ethics (OHSN-REB# 20170829-01H).

### Identification of participants

Adult English-speaking individuals with or without symptoms of URTIs presenting to a mixed urban/rural family practice in Eastern Ontario, Canada were eligible to participate in this study. We did not specifically seek individuals who were at the clinic due to URTI, given that the majority of (if not all) adults will have had previous experience of having a URTI. Individuals who were younger than 18, or who could not speak English, or had severe pain or illness were excluded.

A family physician working in the practice (AA) identified potentially eligible individuals through convenience sampling and gave them a postcard with study information and the contact information of the researcher (SM). Individuals willing to be interviewed were asked to meet with the researcher who was available in the clinic. The researcher provided further information about the study and stressed that participation was voluntary and would not affect the care they would receive during their current or future visits. The researcher also provided them with a consent form. Once written consent was received, a telephone interview was scheduled for a time convenient for the participants.

### Interview guide

The interview guide was designed in collaboration with a health psychologist with expertise in behavioural theories (AMP). It included demographic questions about gender, age and level of education, as well as open-ended questions related to individuals’ experiences with URTIs which were based on CS-SRM dimensions (see Additional file 1). The interview guide was piloted with three individuals to ensure all dimensions of the CS-SRM were adequately covered and questions were clear for participants. We changed the interview guide accordingly to improve the quality of data collection.

### Interview procedure

All interviews were conducted by one researcher (SM). At the start of the interview, confirmatory verbal consent was sought for participation and recording of the interview. SM explained that the study aimed to understand individuals’ perceptions and experiences about URTIs. Participants were informed that they could skip a question if they felt uncomfortable answering it and that they could stop the interview anytime if they were no longer willing to participate in the study. SM also explained the definition of URTIs to the participants. Since it was a semi-structured interview, the participants were given the opportunity to elaborate on any of the questions. SM probed further when answers seemed unclear or short, and the participants were willing to talk more about the issue. The interviews were ended when both the interviewer and the interviewee felt comfortable that all the questions were discussed. Recruitment continued until data saturation in our setting was achieved (i.e. until three consecutive interviews did not add additional concepts/ideas) [29].

### Data analysis

Interviews were transcribed verbatim and anonymized. Common to all qualitative research is the influence the researcher who is conducting the study may have on the analysis. This can have both positive and negative consequences. A researcher knowledgeable in the area under investigation will allow for richer interpretation of the data, but this can also lead to unintended biases. To help balance the potential limitation we included two coders in the analysis (one who conducted the interviews and has content knowledge (SM) and one with less content knowledge but who was knowledgeable in the CS_SRM (AMP)). In addition, data analysis and interpretation were reviewed by the larger research team who were not so closely imbedded within the data. Two coders (SM, AMP) independently reviewed the interview transcripts and coded them using NVivo 10 software [30]. Thematic analysis related to the dimensions of CS-SRM was applied [16]. A combination of inductive and deductive approaches was used. The CS-SRM dimensions provided an initial coding scheme. Since coping strategies are not specified in the CS-SRM, the coders developed different subcategories of these strategies by reviewing all the transcripts. This resulted in identifying four major subcategories: 1) visiting a doctor, 2) using antibiotics, 3) problem-focused coping and 4) self-management. Sources of information was added as a sub-theme to all four subcategories if applicable, to understand what sources of information were used to help the individual to develop that specific perception or behaviour. The coders met after coding the first transcript to compare their results and develop a uniform scheme for coding the quotes. All discrepancies were discussed, and consensus reached. The reliability between the two coders was assessed by an inter-rater reliability coefficient (kappa score). This measure was used to report coding consistency within the CS-SRM and ensure that coders had a good understanding of the dimensions.

## Results

We received the contact information of 30 people and interviewed until data saturation. Saturation was achieved after 15 interviews. The individuals (six females, nine males) were interviewed by telephone, of whom four were parents of dependent children. Parents shared their own personal experiences as well as their experiences as parents of children with URTIs. All except one participant had attended the family practice for reasons other than URTIs. The interviews took between six and 36 min (median: 12). The participants were between 18 and 72 years old. The highest level of education was high school in five individuals, community college in four individuals and university education in six individuals.

The kappa score ranged between 0.23 (for prevention) and 1.00. However, in 85% of the coding, the Kappa score was higher or equal to 0.8.

### Cognitive illness representations

#### Identity

When asked about their symptoms of URTIs, participants often described cough, runny or stuffed nose, post-nasal drip, sore throat, headaches and body aches. Many participants also noted that symptoms tended to be mild, although occasionally more severe symptoms (e.g. prolonged cough) could occur. One individual believed that their URTI symptoms were changing over time and their URTIs now lasted longer than before.

*“Mine are usually cough, nasal, I would just say that. I don’t get anything really serious.” (P9).*

*“I feel there is a tickling in my throat. There’s some kind of a taste that is not right…I say oh, that looks like there is a cold just coming.” (P11).*

#### Timeline

Most participants noted that they usually experienced URTIs at least once a year. There were a few participants that reported having URTIs more (two to three times a year) or less often (every three to four years). Participants reported that the symptoms would typically last between three to 14 days; however, one individual stated that the cough often lasted for a couple of months.

*“Not very long. My immune system must be pretty good, probably just a couple of days.” (P14).*

*“I’m functional in a week, but I’m not over it for 10 days to two weeks.” (P15).*

#### Cause

A variety of causes were mentioned by participants; close contacts with sick people at public places or kids at school, low immune system or being more susceptible, not washing their hands (especially after touching door knobs or flushing the toilet), not eating healthily or drinking enough water, season changing, cold weather and stress.

*“So, it was end of the year, a very stressful time period for me, so everything went up in the air and so just my body said OK enough, I can’t deal with this, you’re going down now.” (P7).*

Viruses and bacteria were stated as cause only by three people. Three participants declared that they really did not know what causes these kinds of infections.

*“Maybe your immune system’s low or, bad bacteria in your system or ... it’s just I don’t know anything causes it.” (P6).*

#### Consequences

The major concerns voiced by many participants were not being able to sleep or work. Other consequences mentioned included cough, body aches, being tired and low energy and difficulty breathing. Another negative consequence of URTIs was not being able to see friends or enjoy trips when travelling. One participant who had a history of bronchitis was afraid that the URTI would turn into pneumonia or bronchitis.

*“The most area it affects you is the mobility to go under full speed and to work.” (P1).*

#### Curability/controllability

Most participants believed that URTIs would last for a period of time (i.e. a few days), eventually go away on their own and that there was no need for antibiotics or prescription medications. Some individuals used over the counter (OTC) medications or home remedies to control the symptoms.

*“You just have to put up with it because you know it’s gonna take its time and then it’s gonna get better.” (P11).*

*“A cold cannot be treated by any modern medicine. All you can do is control the symptoms so that you don’t feel like you’re dying.” (P7).*

One of the participants noted changes in their management as they age.

*“I guess as I get older, things get tougher to fight off*.*” (P5).*

One of the participants described their cough as uncontrollable. Another participant rationalised the use of antibiotics to manage URTIs:

*“I don’t really know, because bacteria need to be treated by antibiotics so, probably there is no other way except for antibiotics.” (P1).*

#### Prevention

Washing hands and staying away from sick people were primarily reported as ways of avoiding URTIs. In addition, eating healthy food and drinking lots of fluid, taking vitamins, proper sleep and being active were considered major preventative measures for URTIs by most participants. Taking flu shots, staying warm, taking raw garlic, and wiping things with alcohol were also mentioned by a few participants.

However, one participant was strongly insistent on the importance of knowledge.

*“Knowing what can and cannot cause it. Knowing what can prevent it. Knowing what you shouldn’t be doing; i.e. going to work and spreading it around things like that. Having that knowledge and following through with the knowledge is, I think at this point in time the best prevention.” (P7).*

### Participants’ sources of information regarding cognitive illness representations

Sources of information on the cause of URTIs were discussed in six interviews; life experience of having previous episodes of the disease, television, internet and family members who worked as doctors or nurses were the main identified sources.

*“I’m 72 years old. I have a lot of experience in life. I have all kinds of other situations I’ve been dealing with. It started when I was a kid. My mum, you know, used to tell me about how to take care of things.” (P11).*

*“I’m familiar enough with my body. I’m comfortable enough knowing what I can and cannot fight off on my own.” (P7).*

Sources of information on curability/controllability of URTIs were mentioned in two interviews; one individual said she used the methods that her mother used to apply to deal with URTIs for herself and her kids and preferred not look things up online. Another participant found booklets in doctors’ offices helpful for this matter.

### Emotional illness representations

Most of the emotional illness representations centered around fear of the URTIs becoming something more severe. One of them found the infections very terrifying, especially if the symptoms were not manageable.

*“If the fever is not manageable, if I can’t manage with Tylenol or Ibuprofen, I am afraid. I don’t want to wait [for a few days before visiting doctor]. I don’t want to be at night, no, no, no.” (P1).*

*“So yeah, definitely a fear of it maybe turning into something more than just a common cold.” (P2).*

*“It’s just damned irritating, that’s all.” (P5).*

Parents felt more concerned when their children showed the symptoms.

*“I’m more, you know, you’re concerned about your children than yourself.” (P14).*

### Coping strategies

Participants reported a number of coping strategies that they used to manage URTIs (Table 1). These included self-management, problem-focused coping, visiting the doctor and using antibiotics.

**Table 1:** Participants’ coping strategies toward URTIs

	Themes (number of individuals)	Quotes
**Self-** **management**	• Used OTC medications (13) • Took a lot of water (4) • Took warm liquids such as tea with honey or lemon and soup (6) • Took multivitamins or Vitamin C (2) • Took more rest or sleep (5) • Gargled water with salt (1) • Took fresh air (1)	*“I just drink a lot of water and try to get a lot of rest and not do too much and they usually go away; it’s pretty quick.” (P2)* *“I just go home, crawl in bed and wait ‘til it’s over.” (P3)* *“I drink an awful lot of water; an awful lot of liquids like especially warm liquids that make you feel better.” (P4)*
**Visiting a doctor**	• Expected to receive a physical examination (4) • Sought a prescription for inhaler (3) • Sought an explanation for getting worse (3) • Asked if antibiotics are needed (2) • Interested to know doctor’s opinion (1)	*“I’m going to have his assessment as to whether it’s an infection or just an ordinary cold.” (P15)* *“Well honestly I’m hoping that there’s nothing wrong with me, but sort of an explanation of why the symptoms are getting worse; if my doctor can figure that out or give me like a reason why they were getting worse.” (P2)* *“I kind of wait a couple of days like 4–5 days. If I’m not feeling better, then I go to a doctor and have them listen to my lungs.” (P4)* *“Doctor at least can look at me and listen to my lungs and can tell me if it’s viral or if it’s bacterial.” (P1)*
**Problem-focused coping**	• Getting a refill for an inhaler (2) • Asking if antibiotics are needed in case of green secretions from nose or throat (2) • Having difficulty managing the fever with Acetaminophen/Ibuprofen (2) • Long-lasting cough (4)	*“He can’t do anything for a general cold but, but if you get an infection in your chest, they give antibiotics for that. So, I’m going to have his assessment.” (P15)* *“I have a puffer at home, if I need a refill, I ask the doctor for a new prescription and it usually helps with the coughing” (P4)* *“I take the cough syrup and do my best and if it continues and I can’t manage it then I go to the doctors” (P8)*
**Using antibiotics**	• Believed antibiotics are not needed for URTIs (9) • Would ask for antibiotics if the symptoms became severe (4) • Used left over antibiotics (1)	*“I always ask if it’s necessary to get antibiotics. If I don’t need, that’s right I, I’m happy and I will take care of myself.” (P1)* “*In my case because it gets into my chest, if I have any prophylactic, if I have any Amoxicillin or something left over from the last course which I know I’m not supposed to do I’ll take that. That doesn’t treat it, doesn’t make it go away faster, but it makes it feel better. It’s not a good thing to do for 2 reasons: 1) it’s probably not helping my cold, 2) I should have taken the full course of medication he gave me the last time.” (P15)*

#### Self-management

Most participants stated that they tried to manage the symptoms by themselves, especially for the first few days of the infection or when it was not very severe. OTC medications such as acetaminophen, ibuprofen, cough drops or tablets, cough syrups, echinacea and nasal drops were widely used. The medications were used as soon as the participants felt the symptoms.

#### Visiting a doctor

Almost all participants mentioned that they did not routinely visit a doctor because of an URTI or at least not for the mild ones. They tried to manage it themselves. But they would consider going to doctor if the symptoms got worse, lasted longer than a few days (three to seven days, depending on the individual), if they had a high fever or problems breathing or swallowing. A few participants considered green secretions from nose or throat as a sign of getting worse.

*“You feel it starts to go to the chest, you start to cough, or maybe green stuff is coming out of your nose then, then that’s when I go and see a doctor and ask for antibiotic.” (P11).*

*“If it’s green and it’s hung on for a couple of days then I’ll take my kids to see the doctor.” (P14).*

A few participants insisted that they usually did not go to the doctor, unless they suspected a more serious illness.

*“If I’m going to the doctor there is something there definitely.” (P7).*

Only one of the participants stated that they would not wait for a few days before visiting a doctor, and would prefer to go sooner than later, so the infection wouldn't get worse. Participants (four individuals) were more concerned when it came to their children; they would take them to the doctor soon after the symptoms appeared or would wait for a maximum of two days before taking them to doctor. One individual mentioned that if she could not manage her children’s fever with OTC medications, she would take them to the doctor.

The participants who did not consider going to doctor, identified different reasons for not going. Some believed it would be a waste of time or money. One individual mentioned not having access to her doctor as the main reason for not going to the doctor.

*“You don’t need a doctor to tell you if you have a sore throat or go get throat lozenges.” (P6).*

When visiting a doctor because of URTIs, participants’ expectations varied from receiving a physical examination (including listening to their lungs), to seeking a prescription for an inhaler or puffer for their cough (Table 1). Most participants stated that they trusted their doctors and accepted their opinions; only one individual mentioned that they would seek a second opinion if their symptoms were interrupting their daily activities and they were not satisfied with the first doctor’s response.

#### Problem-focused coping

There were a number of specific problems that would take the participants with URTIs to the doctor. These included: getting a refill for an inhaler/puffer for the cough, asking if antibiotics were needed in case of green secretions from nose or throat, having difficulty managing the fever and a long-lasting cough (more than two weeks). All these participants mentioned that otherwise they would manage URTIs themselves without going to a doctor.

#### Using antibiotics

Most participants stated that they did not use antibiotics for URTIs, and they believed that antibiotics were not needed for URTIs. Some individuals mentioned that they would ask for antibiotics if the infection got into their chest or if their symptoms were interrupting their daily activities, especially for their children. However, they stated that at the end they would accept the doctors’ opinions even if no antibiotics were prescribed. One individual revealed that they used leftover antibiotics from previous episodes, because they believed that the antibiotic helped to clear up their cold pretty quick, even though they knew that they were not supposed to do that.

Reasons for not using antibiotics for URTIs included concerns around bacterial resistance (mentioned by five individuals) and the fact that the antibiotics killed the good stuff in the gut (four individuals).

*“Not everything requires an antibiotic. Too many being prescribed, too many antibiotics on several occasions each year, you’re growing a resistance to them.” (P4).*

*“The more that people use them (antibiotics), the more the bugs are gonna get resistant which is probably a huge issue in the broad spectrum the next 20 years.” (P15).*

*“If you take too many (antibiotics), it’s not good. Because it strips your good bacteria from your stomach, and can I guess it makes you sicker.” (P13).*

A noticeable number of participants (six individuals) did not know the side effects of using antibiotics and a few of them mentioned that their doctors did not talk to them about the side effects when prescribing an antibiotic. The doctors mostly would be described as emphasizing the need to take the antibiotics with food and completing the course of treatment.

### Participants’ sources of information regarding coping strategies

Participants reported that they got the information from Internet (e.g. WebMD [31]), TV, Telehealth and pharmacists. Regarding antibiotics and their side effects, most participants relied on pharmacists, medications package inserts or Internet; only one individual mentioned that they would ask their questions from the doctor.

*“The expert on any form of medication is always a pharmacist.” (P4).*

Some individuals put emphasis on their lifelong experiences or common-sense, as well as the things they learnt from their mothers.

*“Because you’ve done it before, gone through the process before with doctors you just might as well just do it yourself.” (P4).*

## Discussion

### Main findings of study and comparison with previous literature

This study explored individuals’ perceptions and beliefs about URTIs and the way they managed them. Although the participants had different beliefs about the causes of URTIs, they generally knew how to manage their symptoms and prevent the infections from occurring. For the most part, individuals applied the same perceptions and coping strategies whether they themselves or their children were ill. Yet, they mentioned that they would be more concerned when it came it to their children.

Almost all participants mentioned that they would not routinely go to the doctor because of URTIs; instead, they tried to manage the symptoms by using home remedies and OTC medications which is consistent with findings from studies in the USA and UK [32, 33]. However, visiting the doctor was often reported as the first choice for many patients with URTIs in studies from South Korea, Malaysia and Qatar [34–36]. This may be in part due to lack of public knowledge about self-management of URTIs [34]. Also, cultural beliefs in some parts of the world, such as belief in the effectiveness of treatments that are received from doctors, could explain these results [36].

Almost all participants mentioned that they would only go to the doctor if there was a serious problem (e.g. breathing or swallowing difficulties, high fever, long-lasting cough). Findings from a study in the Netherlands also reported that the small number of patients, who decided to visit doctors, often had good reasons (e.g. serious symptoms, suffering for more than two weeks, respiratory comorbidity) [37].

When visiting a doctor, participants mostly wished to be examined and to gain an explanation for their symptoms. Consistent with our results, thorough examination, explanation and reassurance were expected by patients with URTIs in USA, UK, South Korea, Germany, Qatar, Denmark and Netherlands [9, 24, 32, 35, 37–39]. Interestingly, some participants reported that their reason for visiting the doctors was to get a refill for inhaler/puffer. URTIs can trigger long-lasting coughs in those who have a history of asthma, or other reactive airway diseases. Those patients would require inhalers because of the cough caused by URTIs.

Our results showed that participants did not usually visit the doctor to ask for antibiotics, which is consistent with other studies [34, 35, 39, 40]. Specifically, a study investigating patients in six European countries with respiratory tract infections revealed that only 2% of these patients explicitly requested antibiotics [40]. Similar findings were reported from other parts of the world (UK, South Korea, Australia, China, Qatar, Denmark, Germany, USA) [24, 34, 35, 38, 39, 41–44]. A study from USA reported that patients put a lot of pressure on doctors for the prescription of antibiotics for URTIs by the way they presented their symptoms. However, they observed that only 6% of cases made direct requests for antibiotics [7]. Another study from USA argued that doctors felt a pressure to prescribe antibiotics from the patients who suggested a candidate diagnosis, but the authors noted that an overt demand for antibiotics was unusual [44]. Conversely Dosh et al. from USA reported that 60% of patients expected antibiotics [6], but their study included respiratory infections for which the antibiotics were sometimes necessary. In addition, these three studies are older compared to other studies and this may in part explain the difference in results. This is consistent with the results of a recent systematic review that showed that the trend of patient expectation for receiving antibiotics for respiratory tract infections is declining over time on a global level [45]. Public knowledge and beliefs may have changed in recent years because of easy access to different sources of information through internet or media.

Most participants believed that there was no need for prescription medication or antibiotics and the symptoms would go away by themselves after a few days. Some participants also mentioned their concerns about time or money as reasons for not visiting their doctors. In Ontario, visiting the primary care provider is covered and people do not pay for consultations out of pocket. However, they may need to pay for their prescriptions. Furthermore, visiting a doctor may require paying for transport/parking, taking day off from work, paying for a babysitter to look after the kids while they are visiting their doctors. Even if the illness is serious and needs to be seen by a doctor, the patients may end up waiting for hours in their doctors’ offices or walk-in clinics. So, some participants preferred to mange their symptoms themselves, instead of spending time waiting in doctors’ offices.

Participants’ strategies for managing URTIs were based on their previous experiences of these infections, common sense and things that they had learnt from their parents in childhood. They mostly relied on family members, Internet and pharmacists as sources of information.

### Implications for research and practice

Our results showed that individuals with URTIs did not necessary ask for antibiotics, instead they expected a thorough examination and an explanation for their symptoms. Although we did not interview the healthcare providers in our setting, the literature review from different settings suggest that some doctors perceive pressure from patients to prescribe antibiotics [6–8, 12, 46–49]. This suggests that there may be a miscommunication between patients and healthcare providers. We believe that better communication with patients could help doctors to elicit patients’ expectations and potentially reduce unnecessary prescriptions while increasing patients’ satisfaction with being heard by their healthcare professionals. Choosing Wisely Canada (CWC) in recent years has tried to promote the conversations between doctors and patients about treatment expectations. As part of their framework, they have encouraged patients to be more engaged in clinical encounters and ask questions about the necessity of treatments or procedures. CWC has also persuaded doctors to change their practice styles and to be more explicit about their clinical decisions with patients [50]. They have just recently published a toolkit for ‘Using antibiotics wisely’ for the management of URTIs in primary care, in which they discuss different ways to change current practice [51]. Furthermore, some useful resources have been developed in other parts of the world, which can be adapted to Canada settings. A good example of these open access resources is TARGET learning series, in which one webinar discussed managing patient expectations among other topics [52].

Individuals in our study had different perceptions about the causes of URTIs. Providing information (by healthcare providers or mass media) that viruses are the main cause of most URTIs may help patients feel more comfortable about not visiting doctors or taking antibiotics for these infections. Furthermore, there were misconceptions among some patients that changes in symptoms might require antibiotics (e.g. if the color of sputum changes to green, antibiotic is needed). However, there is evidence that these changes do not imply the need for antibiotics [53, 54]. When patients are visiting their doctors because they are concerned that due to their symptoms, they need antibiotics, the doctors could use those opportunities during patient visits to clarify the reasons for patients’ concerns, as well as addressing those concerns with evidence-based information.

Our results were based on English speaking individuals’ beliefs and perceptions in a single practice. However, Canada consists of individuals from different ethnicities and cultural backgrounds. Studying individuals from different locations or ethnicities may identify new concepts that are specific to those groups.

### Strengths and limitations of the study

Our study has identified a number of factors that could be addressed by interventions to reduce antibiotic use. Some of our findings confirm those of previous studies, but this study allowed us to better understand this issue in our context and explore how our context differs from other settings.

Except for a few studies that have used grounded theory model [32], Theory of Planned Behaviour [33] and Andersen’s behavioural model [34], most studies have not used any theoretical models in their investigations to elicit patients’ perceptions of URTIs [e.g. 24, 35, 36, 41, 42, 43]. To our knowledge, this is the first qualitative descriptive study to apply a theory-based approach to study this topic in the Canadian context. Regardless of the results, we believe that using a theory can help in guiding the research process and reducing researchers’ possible biases in interpreting and analysing the findings. Also, its pre-defined constructs, facilitate the design of possible interventions, by identifying behavioural strategies that specifically target the constructs identified as contributing factors to requesting antibiotics, to be implemented in an intervention.

Although the CS-SRM is rarely used for studying acute diseases, it showed to be very useful in demonstrating individuals’ perceptions (illness representatives) of URTIs. This is of great importance with regards to designing future interventions to reduce unnecessary use of antibiotics for URTIs. Because this allows us to focus our interventions on those specific illness representatives which may not be based on scientific evidence, and by changing individuals’ illness representatives over time, we can expect to change their behaviours.

While our study presented a novel perspective on determinants of patients’ behaviors, it had a few limitations. By confining our sample to English speaking individuals, we may have missed varying perceptions from other patients. In addition, we used convenience sampling and approached the individuals who were available in the clinic and willing to participate. Although most individuals who were approached at the clinic agreed to participate in the study, we do not know if those who did not participate were different from our sample regarding their beliefs and behaviours about URTIs. Furthermore, all except one of the participants did not have symptoms of URTI at the time of interview. This allowed us to recruit more participants in a shorter time period. URTIs are very common in the society, and even if the participants did not have any symptoms at the time of the interview, they have had experienced it before, and could tell us about their perceptions and experiences. Therefore, those interviews relied on participants’ past experiences and may have been affected in part by recall bias. Despite these limitations, our findings were supported in part by other studies that investigated patient perceptions about managing URTIs and provided valuable insight into improving patient-physician communication to improve self-management of URTI symptoms and reduce antibiotic prescribing.

## Conclusion

Participants had a good understanding about URTIs. Most participants tried to manage the symptoms by using OTC medications, especially for the first few days of the infection or when it was not very severe. They did not usually go to doctors because of URTIs, and if they went, most of the times they did not seek antibiotics, but instead they wanted to be examined and receive an explanation for their symptoms.

## Supplementary information

**Additional file 1.** Interview guide.

## Data Availability

The datasets generated and/or analysed during the current study are not publicly available due to lack of consent from the participants to share the raw data from their interviews to anyone other than the research team but are available from the corresponding author on reasonable request.

## Abbreviations

AA: Alykhan Abdulla
AB: Antibiotic
AMP: Andrea M. Patey
COPD: Chronic obstructive pulmonary disease
CS-SRM: Common Sense-Self-Regulation Model
CWC: Choosing Wisely Canada
DS: Dawn Stacey
JMG: Jeremy M. Grimshaw
OTC: Over the counter
RSB: R. Sacha Bhatia
SM: Sameh Mortazhejri
URTI: Upper respiratory tract infections
