# Supplementary material for: Understanding determinants of patients’ decisions to attend their family physician and to take antibiotics for upper respiratory tract infections: a qualitative descriptive study
Source: BMC Fam Pract. 2020 Jun 24;21:119. doi: 10.1186/s12875-020-01196-9 (PMC7313109; doi:10.1186/s12875-020-01196-9)
Supplement: Supplementary file 1 — Additional file 1. Interview guide. [file 12875_2020_1196_MOESM1_ESM.docx]

**Additional file 1**

**Interview guide**

Thank you for participating in this interview. Information you provide in this interview will be identified by a study ID code and will not be associated with your name or other personal identifying information. The purpose of the interview is to understand your point of view regarding how you manage upper respiratory infections (URTIs) (e.g. common cold, sore throat, sinusitis…). The information you provide here, including your thoughts and comments will not be shared with your family doctor, or other healthcare providers. There’s no right/wrong way to answer the questions; however, we are looking for honest answers. It helps us to understand the current concerns of patients.

I would like to start with some general questions:

1. Gender
2. Your age?
3. Your highest level of education?

Now, I would like to talk to you about your experience with having an upper respiratory infection and how you manage that. When I say URTI, I’m talking about respiratory infections that affect the chest, throat, nose, sinuses and ears. Chronic conditions such as asthma, chronic obstructive pulmonary disease (COPD) or the infections of lower respiratory tract (e.g. bronchitis) are not the scope of this interview.

1. How often do you usually get URTIs? (Timeline)
   1. Do you remember what your last respiratory infection was like?
2. When was it? What were your symptoms like? The duration of your symptoms? The severity of your symptoms? How did it make you feel? (Identity, timeline)
3. What do you think causes URTIs? (Prompts biological causes, emotional causes, environmental causes) (Cause)
4. What are the consequences of URTI for you? (Prompts it may make you less effective at work? You might get complications from the URTI (e.g. chest infection)? What is the worst thing that can happen to you when you suffer from a cold? (Consequences)
5. What is your usual approach to looking after yourself when you have an URTI? Does it work? (Coping strategies)
6. Do you ever look up information about URTIs and how to treat them? If so, where? Under what circumstances (i.e. any symptoms would trigger a search?) Where do you look for information? (E.g. internet, family, friends, doctor) (Sources of information)
7. Do you usually visit a doctor for an URTI? (Coping strategies)

a) If yes,

1. When do you go? (Prompts As soon as the symptoms appear (any symptoms?)? Or it takes you a few days before going to doctor?
2. What are you aiming for when you see a doctor? (Prompts For a prescription? For reassurance? For a referral to a specialist?
3. What factors would make you go see a doctor? (Prompts For example, the severity of the symptoms? The duration of the disease? The friends/colleagues’ advices? The fear of disease complications?
4. In your experience, did visiting the doctor have any effect on your disease?

b) If no,

1. Why? (Prompts because of factors related to your time, your doctor, your disease, the medication)
2. In your experience, has your disease (the duration, the severity) been affected by not seeking a doctor?
3. Are you aware of the pros and cons of taking antibiotics? What sources have helped you to building this knowledge? (Coping behaviors, curability/controllability)
4. Does the doctor usually talk to you about the pros and cons of taking antibiotics?
5. Do you share your concerns with your doctor? (That you think if you don’t get AB, you won’t get better) or (you don’t want AB, because of its side effects…)
6. If you took AB for your last URTI, did it shorten the duration of the disease? Relieved the symptoms?
7. How do you think URTIs can be cured? (Curability/controllability)
8. Do you think that URTIs can be prevented? If yes, how? (Prevention)

Thank you. Is there anything else about your management of URTIs you would like to talk to me about that I haven’t covered in this interview?
